# Polarimetric SAR Time-Series for Identification of Winter Land Use

**DOI:** 10.3390/s19245574

**Published:** 2019-12-17

**Authors:** Julien Denize, Laurence Hubert-Moy, Eric Pottier

**Affiliations:** 1 University of Rennes & IETR UMR 6164, 35 000, Rennes, France; eric.pottier@univ-rennes1.fr; 2 University of Rennes & LETG UMR 6554, 35 000, Rennes, France; laurence.moy@univ-rennes2.fr

**Keywords:** crops, RADARSAT-2, sentinel-1, ALOS-2, C-band frequency, L-band frequency, dual-polarization, quad polarization, random forest classification

## Abstract

In the past decade, high spatial resolution Synthetic Aperture Radar (SAR) sensors have provided information that contributed significantly to cropland monitoring. However, the specific configurations of SAR sensors (e.g., band frequency, polarization mode) used to identify land-use types remains underexplored. This study investigates the contribution of C/L-Band frequency, dual/quad polarization and the density of image time-series to winter land-use identification in an agricultural area of approximately 130 km² located in northwestern France. First, SAR parameters were derived from RADARSAT-2, Sentinel-1 and Advanced Land Observing Satellite 2 (ALOS-2) time-series, and one quad-pol and six dual-pol datasets with different spatial resolutions and densities were calculated. Then, land use was classified using the Random Forest algorithm with each of these seven SAR datasets to determine the most suitable SAR configuration for identifying winter land-use. Results highlighted that (i) the C-Band (F1-score 0.70) outperformed the L-Band (F1-score 0.57), (ii) quad polarization (F1-score 0.69) outperformed dual polarization (F1-score 0.59) and (iii) a dense Sentinel-1 time-series (F1-score 0.70) outperformed RADARSAT-2 and ALOS-2 time-series (F1-score 0.69 and 0.29, respectively). In addition, Shannon Entropy and SPAN were the SAR parameters most important for discriminating winter land-use. Thus, the results of this study emphasize the interest of using Sentinel-1 time-series data for identifying winter land-use.

## 1. Introduction

The importance of vegetation cover during winter to preserve soil quality and water resources is now well-recognized by scientists, decision makers and citizens, and land-use mapping is considered a relevant input into decision-making to implement appropriate policy responses [1]. However, although identifying land use in agricultural areas is a major environmental and scientific issue [2], it remains challenging due to its high spatio-temporal dynamics [3]. In this context, remotely sensed time-series data are a valuable tool to identify land use by providing precise and timely information about the phenological status and development of vegetation at different scales, from local to global extents [4,5]. In the past few decades, progress has been made with the development of high and very high spatial and temporal resolution optical (e.g., Satellite pour l’observation de la Terre (SPOT-6/7), Sentinel-2) and Synthetic Aperture Radar (SAR) (e.g., TerraSAR-X, RADARSAT-2, Advanced Land Observing Satellite 2 (ALOS-2), Sentinel-1) sensors [6,7,8]. However, using optical time-series to identify land use in winter is limited by cloud cover and/or low solar irradiance [9], and late winter is a critical period during which vegetation begins to grow [3]. Conversely, SAR time-series provide a reliable solution to address the limitations of optical images because they are not sensitive to atmospheric conditions and can operate day and night [10].

These advantages, along with a sensitivity of microwave scattering to soil and vegetation characteristics [11,12], have led scientists to evaluate the potential of using SAR sensors to monitor agriculture [13,14,15,16]. The potential of using SAR data to identify land use is based on the sensitivity of the radar signal to the dielectric constant of the objects and to their structure (i.e., the distribution of size shape and orientation of the scatterers) [11,17]. Thus, some studies have demonstrated the ability of SAR backscattering-coefficient and polarimetric data to classify land use using the dielectric properties of soil, surface roughness and vegetation canopy structure [18,19,20,21]. Most of these studies used the C-Band frequency rather than other frequencies (e.g., X- or L-Bands). Since microwave penetration depends on wavelength and incident angle, L-Band (~20 cm) wavelengths penetrate further into crop canopies than those of the C-Band (~5.5 cm). Consequently, C-Band wavelengths interact more with the upper canopy, while L-Band wavelength responses result from greater interaction with the soil/canopy and scattering directly from the soil [11,12]. Thus, McNairn and Brisco [13] demonstrated the potential of vertically (V) and horizontally (H) polarized C-Band microwaves to identify land use. Indeed, since V microwaves respond to predominantly vertical structure, they penetrate the canopy less. Conversely, H microwaves tend to penetrate the canopy more than V microwaves. Similarly, others [17,18] illustrated the ability of the L-Band and the combined use of C- and L-Bands to classify land use. Many studies have also shown the potential of radar data to map crop residues [13,22] or crop types during summer [23,24].

However, only a few studies have demonstrated the potential of SAR time-series to identify land-use types in winter. Haldar et al. [16] illustrated the potential of polarimetric C-Band SAR time-series data to derive useful information, such as biophysical parameters, from mustard (*Sinapis alba L.*) and wheat (*Triticum aestivum L.*) crops. Similarly, few studies have shown Sentinel-1′s potential to identify and characterize land-cover and land-use dynamics. For example, some studies [5,25,26,27] have shown benefits of using Sentinel-1 time-series to understand crop behavior and dynamics and classify land use. Likewise, Minh et al. [28] used Sentinel-1 time-series to produce a winter vegetation quality map with five classes (“bare soil” to “high quality”) based on a deep-learning method, with an overall accuracy (OA) >98%. However, the specific configurations of SAR sensors (e.g., band frequency, polarization mode) used to identify land-use types remain underexplored, and even unexplored for winter land-use.

This study aimed to evaluate and compare the value of multi-temporal ALOS-2 (ALS-2), RADARSAT-2 (RST-2) and Sentinel-1 (S-1) data for monitoring winter land-use in agricultural areas. Specifically, we addressed the main question: What is the most appropriate SAR configuration (polarization, frequency, density of time-series) for characterizing winter land-use? To this end, we first calculated SAR parameters from quad-polarization (pol) C-Band, dual-pol C-Band and dual-pol L-Band time-series to generate several parameter datasets. We then applied a classification procedure using the Random Forest (RF) algorithm to determine the most discriminating SAR configuration for land-use monitoring in winter. Finally, we identified advantages and disadvantages of the method and its results.

## 2. Materials and Methods

### 2.1. Study Site

**Figure 1:**
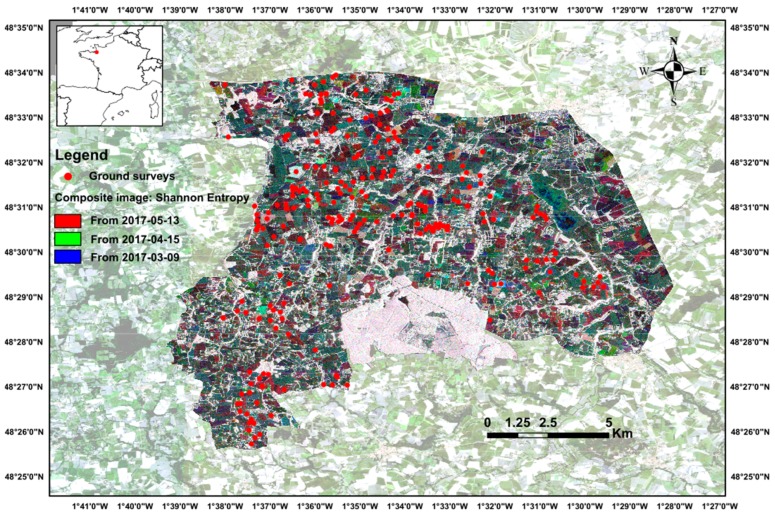
Study site location, ground surveys (RGB composite image constructed from Shannon Entropy extracted from Advanced Land Observing Satellite 2 (ALOS-2) data for three dates: 03-09-2017, 04-15-2017 and 05-13-2017. ©Kalidéos data 2017 and JAXA data).

The study site is a relatively flat area located to the south of the Bay of Mont-Saint-Michel (8° 31′ 0” N, 1° 31′ 30” W) in France and covers an area of ca. 130 km² (Figure 1). This site has been included in long-term ecological research (LTER) networks at the European (LTER-Europe) and international (ILTER) levels since 1993 to assess relationships between changes in farming activities, landscape dynamics and ecological processes related to biodiversity, water quality and climate [29]. This study site is also referenced in the “Kalidéos program”, which is coordinated and managed by the CNES (French Space Agency) to promote and demonstrate the use of spatial data by supporting research and development, prototyping and user demonstration activities. The site is composed of ca. 7 000 agricultural fields ranging from 0.1–65 ha and is characterized by a fragmented agricultural landscape (from a hedgerow network to open fields). This temperate climate area is exposed to mean annual precipitation of 600–700 mm and mean average temperature >12 °C. In summer, farming systems are based on one main crop per field: maize (*Zea mays* L.), wheat (*Triticum aestivum* L.), rapeseed (*Brassica napus* L.) or barley (*Hordeum vulgare* L.). In winter, in addition to grasslands, which play a major role in regulating water flows and nutrient cycling, catch crops are sown to decrease nitrogen leaching, as required by the European Union’s “Nitrates Directive” [30].

### 2.2. Field Data

**Figure 2:**
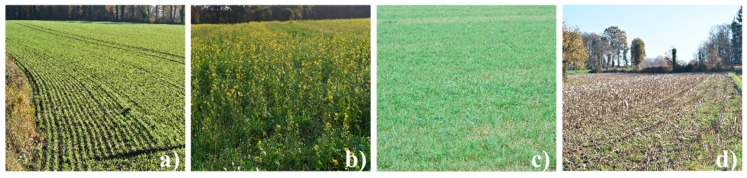
The mainland-use types encountered in winter in the study area: (**a**) winter crops (winter barley), (**b**) catch crops (mustard), (**c**) grasslands and (**d**) crop residues (maize stalks).

The mainland-use types encountered and investigated in winter in the study area (Figure 2 and Table 1) are the following:

- Winter crops, which cover ca. 40% of the UAA (utilized agricultural area) and include three main annual crops: winter wheat, winter barley and rapeseed
- Grasslands, which cover ca. 30% of the UAA and can be mown or grazed
- Catch crops, which are sown after harvest of the main crop from August to October, cover ca. 25% of the UAA and include a wide variety of crops
- Crop residues, which cover ca. 5% of the UAA and correspond to maize stalks that are left in fields when the maize is harvested after 1 November [3]

Field observations were conducted in 231 crop fields monthly from November 2016 to February 2017 to calibrate and validate classification of remote sensing data (Figure 1). Samples were randomly distributed throughout the study site, with half of the fields (116) being used for training and the other half for validation. The fields inventoried ranged in size from 0.1–65 ha. The number of training samples of each winter land-use class inventoried was 54 for winter crops, 14 for grasslands, 42 for catch crops and 6 for crop residues.

### 2.3. Satellite Data

#### 2.3.1. RADARSAT-2 Time-Series

A series of 10 RST-2 SAR images from October 2016 to May 2017 were acquired by MacDonald, Dettwiler and Associates and provided by the VIGISAT project managed by Collecte Localisation Satellites in the framework of the GIS Bretel (“Groupement Bretagne Télédétection”, Bretagne, France) (Table 2). RST-2 images were acquired in Single Look Complex (SLC) mode (delivered in quad-pol mode: HH, HV, VH and VV polarization states) with an incidence angle of 35°. The range and azimuth spatial resolutions were 8.2 and 4.7 m, respectively.

#### 2.3.2. Sentinel-1 Time-Series

Two series of eight and twenty S-1 SAR images, respectively, from August 2016 to May 2017 were acquired by the European Space Agency and provided by its data hub [31] (Table 2). S-1 images were acquired in SLC mode (delivered in dual-pol mode: VH and VV) with an incidence angle of 31° to 46° and an angle of 40° on the study area. The range and azimuth spatial resolutions were 2.3 and 13.9 m, respectively.

#### 2.3.3. ALOS-2 Time-Series

A series of six ALS-2 SAR images from January–June 2017 were acquired by the Japan Aerospace Exploration Agency and provided by the Kalidéos program [32] (Table 2). ALS-2 images were acquired in SLC mode (delivered in dual-pol mode: HH and HV) with an incidence angle of 40° and range and azimuth spatial resolutions of 1.4 and 1.9 m, respectively.

### 2.4. Extraction of SAR Parameters

SAR quad-pol and dual-pol parameters from the three sensor datasets (RST-2, S-1 and ALS-2) were calculated using Sentinel Application Platform (SNAP) v6.0 (Develop by Esa, Paris, France) and PolSARpro v5.1.3 software [33] (Develop by IETR, Rennes, France).

#### 2.4.1. Quad-Pol Time-Series

- Backscattering coefficients ($\sigma^{0}HH$, $\sigma^{0}HV$, $\sigma^{0}VH$, $\sigma^{0}VV$) were calculated from the radiometrically calibrated RST-2 time-series using SNAP according to Equation (1) [34]:
  (1) $$\sigma_{j}^{o}=\;\beta_{j}^{o}+10\times \;\log_{10}\left(\sin I_{j}\right)$$
  where *β* is the radar brightness and $I_{j}$ is the incidence angle at the $j^{th}$ range pixel.
  Equation (1) assumes that the Earth is a smooth ellipsoid at sea level. A Lee Sigma filter [35] was applied with a window of 7 × 7 pixels and a sigma value of 0.8. RST-2 images were then geocoded at an 8-m resolution using Shuttle Radar Topography Mission 3s data to correct topographic deformations. The accuracy of geometric correction was less than 8 m per pixel. Next, two backscattering ratios were calculated (*σ°HH:σ°VV*, *σ°HH:σ°HV*) that highlight scattering mechanisms of each target.
- Polarimetric parameters were calculated from SLC RST-2 time-series. First, a 3 × 3 coherency matrix $T_{3}$ was extracted from the scattering matrix (S) of each image using PolSARpro. Next, a Lee Sigma filter was applied with a window of 7 × 7 pixels and a sigma value of 0.8. The elements of the matrix, which are independent of the polarimetric absolute phase [36], were then geocoded directly using SNAP with an 8-m resolution.

Second, Cloude–Pottier decomposition [37] was then calculated based on the $T_{3}$ matrix. From the eigenvalues extracted, we calculated three independent parameters: (i) entropy (H), which expresses the randomness of the scatter; (ii) alpha angle (α), which describes the dominant scattering mechanism and (iii) anisotropy (A), which represents the relative power of the dominant mechanism. In addition, four parameters based on the Cloude–Pottier decomposition (H×A; H×(1−A); (1−H)×A; (1−H)(1−A)) were calculated because they can provide the number of scattering mechanisms in each resolution cell.

Third, Freeman–Durden decomposition [38] was used to model the 3 × 3 covariance matrix ($C_{3}$) as the contribution of three scattering mechanisms for each pixel: volume, double-bounce and surface/single-bounce. Fourth, SPAN (total scattered power) and Shannon Entropy (SE), which equals the sum of two parameters related to the intensity ($SE_{i}$) and degree of polarization ($SE_{p}$) [36], were calculated from the $T_{3}$ matrix. SE measures the disorder encountered in polarimetric SAR images.

Finally, two polarimetric parameters were extracted from the $T_{3}$ matrix: pedestal height and the Radar Vegetation Index (RVI). Pedestal height is the ratio of the maximum received intensity to the minimum received intensity; it indicates the presence of unpolarized scattering and thus the degree of polarization of a scattered wave [39]. RVI is a function of incidence angle, since the path length through the vegetation canopy will increase as the incidence angle increases [40]. Ranging from 0 to 1, RVI measures the randomness of the scatter according to Equation (2):

(2) $$RVI=\frac{8\sigma^{0}HV}{\sigma^{0}HH_{\;}+\sigma^{0}\mathrm{VV}\;+\;2\sigma^{0}HV}$$

A total of 25 quad-pol parameters were calculated for each of the 10 quad-pol 8-m RST-2 images, yielding a dataset with 250 variables.

#### 2.4.2. Dual-Pol Time-Series

The dual-polarization preprocessing step included (i) conversion of RST-2 time-series images from quad- to dual-polarization mode, (ii) resampling of RST-2 and ALS-2 time-series images at the spatial resolution of S-1 images and (iii) extraction of dual-pol C-Band and dual-pol L-Band SAR parameters.

- *Converting the RST-2 time-series polarization mode*: Each 3 × 3 coherency matrix $T_{3}$ extracted from RST-2 quad-polarization images was converted to a 2 × 2 covariance matrix $C_{2}$ using PolSARpro. The converted RST-2 images had the same polarizations (HH and HV) as ALS-2 images.
- *Calculating S-1 and ALS-2 covariance matrices*: A 2 × 2 covariance matrix ($C_{2}$) was extracted from the two polarizations of each ALS-2 2-m image and each S-1 image (for S-1 dense and sparse time-series).
- *Resampling RST-2 and ALS-2 time-series*: A multi-looking function was applied using a 2 × 1 pixel window for RST-2 images (i.e., 2 × 4.7 m and 1 × 8.2 m) and a 5 × 6 pixel window for ALS-2 images (i.e., 5 × 1.9 m and 6 × 1.4 m) using PolSARpro. Resampled RST-2 images had a resolution of 9.4 × 8.2 m, which was close to that of resampled ALS-2 images (9.5 × 8.4 m) and 10-m corrected S-1 images.
- *Calibrating backscattering coefficients*: Backscattering coefficients σ°HH and σ°HV were simultaneously calibrated radiometrically from dual-pol converted and resampled RST-2 images and original and resampled ALS-2 images using SNAP. Backscattering coefficients σ°VV and σ°VH were simultaneously calibrated radiometrically from dual-pol S-1 images. Then, a Lee Sigma filter [35] with a window of 7 × 7 pixels and a sigma value of 0.8 was applied to all images to attenuate speckle noise. Next, geometric correction was performed using the Shuttle radar topographic mission (SRTM) for each time-series dataset, with a 2-m resolution for original ALS-2 images; 8-m resolution for original RST-2 images and 10-m resolution for resampled ALS-2, resampled RST-2 and the two S-1 images. Finally, the σ°HH:σ°HV ratio and σ°HH−σ°HV difference were calculated from ALS-2 and RST-2 backscattering coefficients, and the σ°VH:σ°VV ratio and σ°VH−σ°VV difference were calculated from S-1 backscattering coefficients.
- *Extracting polarimetric parameters*: Dual-polarimetric parameters were simultaneously extracted from dual-pol S-1 images, converted and resampled RST-2 images and original and resampled ALS-2 images. To this end, the same Lee Sigma filter was applied to the $C_{2}$ matrices to filter out speckle noise. Then, geometric corrections were applied to all polarimetric parameters using the SRTM with the previously used 2-m, 8-m and 10-m resolutions. SPAN, SE, SEi, SEp, normalized SE, normalized SEi and normalized SEp were also extracted.

A total of 11 dual-pol parameters (2 backscattering coefficients, 1 ratio and 1 difference based on the backscattering coefficients, 1 SPAN, 2 SE, 2 SEi and 2 SEp) were calculated for each of the 10 RST-2, 6 ALS-2 and 8 or 20 S-1 images. Finally, six dual-pol datasets were created:

1. original RST-2 8-m dataset with 110 variables (11 parameters × 10 dates)
2. resampled RST-2 10-m dataset with 110 variables (11 parameters × 10 dates)
3. original ALS-2 2-m dataset with 66 variables (11 parameters × 6 dates)
4. resampled ALS-2 10-m dataset with 66 variables (11 parameters × 6 dates)
5. sparse S-1 10-m dataset with 88 variables (11 parameters × 8 dates)
6. dense S-1 10-m dataset with 220 variables (11 parameters × 20 dates)

### 2.5. Classification of SAR Parameter Datasets

A two-step approach was performed to identify winter land-use using the seven SAR parameter datasets (one quad-pol and six dual-pol). First, parameter importance was analyzed for each SAR parameter dataset using the RF importance function, The RF importance function was performed to rank the features in order of importance based on the mean decrease in the Gini index (Breiman et al., 2001) repeated 100 times by changing the training sample, to identify the parameters most important for identifying winter land-use types. A measure of variable importance was provided for each candidate predictor and each classification using the heuristic method based on the Gini Index [41,42,43].

Second, RF, as a supervised classification algorithm, was used to classify land-use during winter 2016–2017. The RF was chosen for its high performance and accurate classification of land use [44]. RF is an ensemble classifier that uses classification and regression trees to make predictions [42]. The trees are created by drawing a subset of training samples through replacement (a bagging approach). In this way, some samples may be selected several times, while other samples may not be selected at all. The “randomForest” package (v.4.6–14) developed by [45] and implemented in R software (v.3.5.1) [46] (Develop by Bell Laboratories, Murray Hill, New Jersey, United-Sates) was used to perform classifications. Two RF parameters were tuned using the “name” function in this package. The first, the number of trees randomly created using the training dataset, was set to 1000, since the number of errors decreases little with more than 1000 trees [47]. The second parameter, the number of variables randomly sampled as candidates at each split node (mtry), was defined starting with a mtry equals the square root of the number of input variables, then searching for the optimal value to improve the quality of the model.

From each image (one per date), 1200 samples (pixels) were randomly selected. Half (600 “in-bag” samples) were used to train the trees, while the rest (600 “out-of-the-bag” samples) were used to estimate the accuracy of the RF model [48]. This process being repeated 100 times with replacement of samples, we can consider that bagging was used during the overall process [3]. Classification accuracy was assessed using the F1-score and the Kappa index. The F1-score is a standard measure of classification accuracy defined as the weighted average of precision and recall [49], while the Kappa index expresses the proportional decrease in error generated by the classification compared to the error of a completely random classification [50].

A four-step classification procedure was performed to evaluate the potential of SAR time-series to identify winter land-use types:

1. RST-2 quad-pol, S-1 dense and ALS-2 2-m time-series datasets were classified to demonstrate the full potential of these SAR sensors
2. RST-2 10-m dual-pol, ALS-2 10-m and S-1 sparse time-series datasets were classified to identify the best band frequency
3. RST-2 quad-pol and RST-2 8-m dual-pol time-series datasets were classified to identify the best polarization mode
4. S-1 dense and sparse time-series datasets were classified to evaluate the influence of the number of images

## 3. Results

### 3.1. Importance of SAR Parameters for Discriminating Winter Land-Use

**Figure 3:**
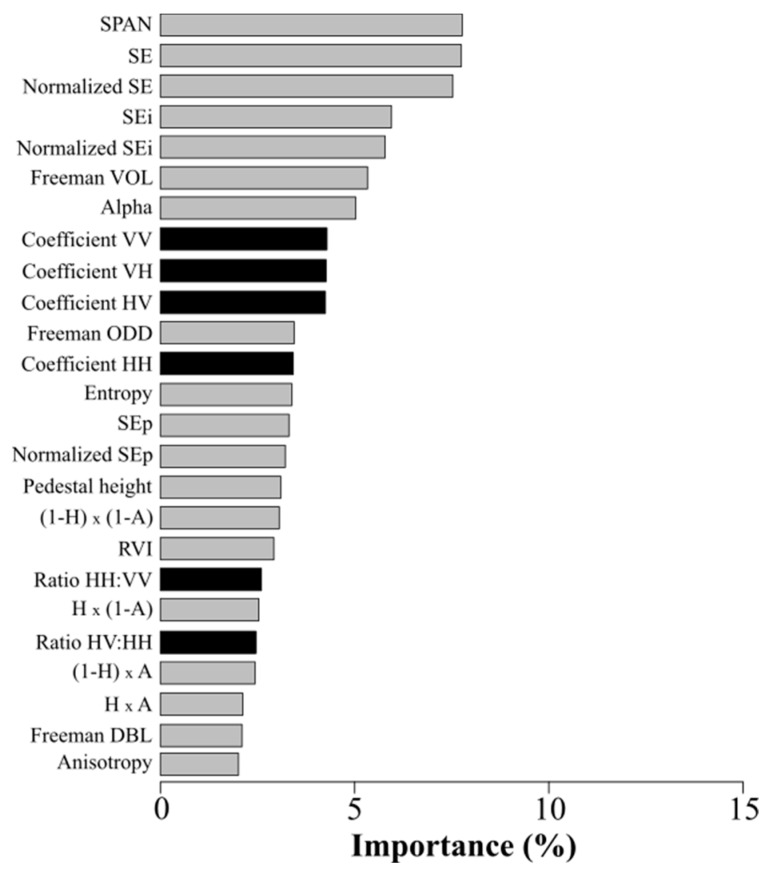
Importance (in %) of quad-pol SAR parameters based on 100 random forest classifications. Parameters related to backscattering coefficients are in black, while polarimetric parameters are in gray. SE: Shannon Entropy.

**Figure 4:**
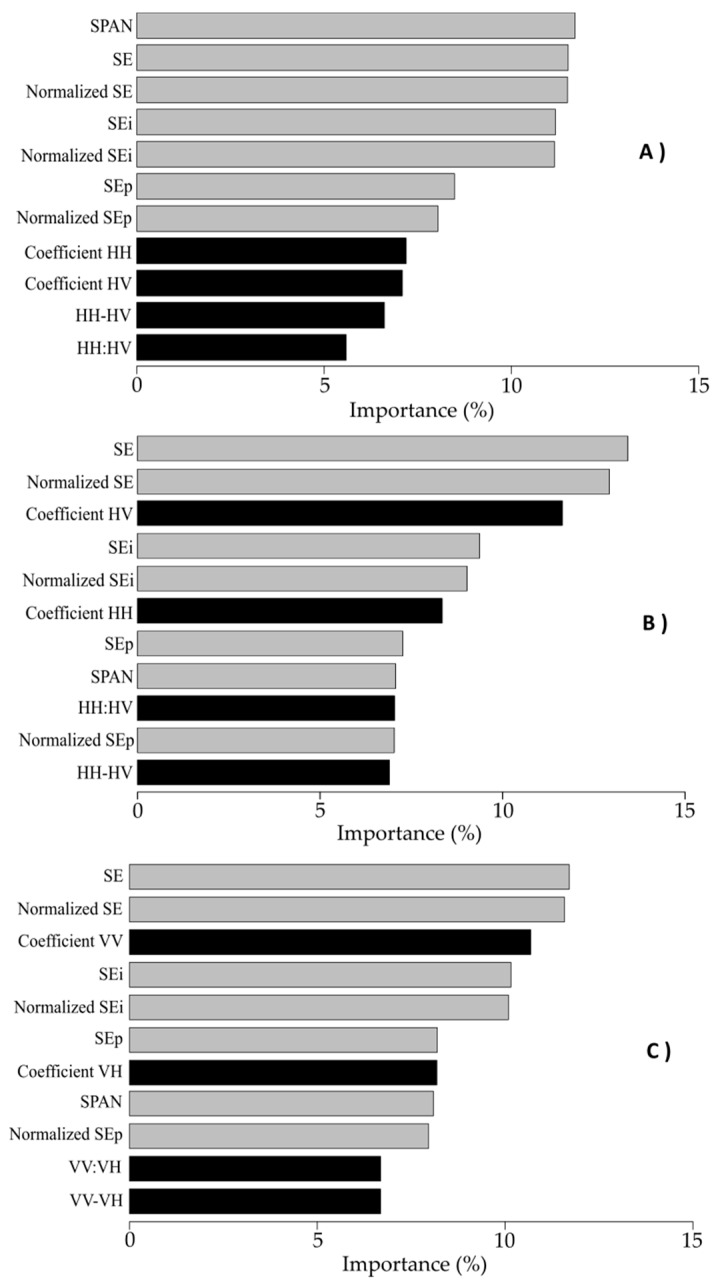
Importance (in %) of dual-pol SAR parameters based on 100 random forest classifications using (**A**) ALOS-2 parameters, (**B**) RADARSAT-2 parameters and (**C**) Sentinel-1 parameters. Parameters related to backscattering coefficients are in black, while polarimetric parameters are in gray. SE: Shannon Entropy.

The most important SAR parameters for discriminating winter land-use depended on the polarization mode and band frequency (Figure 3 and Figure 4). Concerning polarization mode, the most important parameters in the quad-pol data were SPAN (7.8%), SE (7.7%), normalized SE (7.5%) and, to a lesser extent, SE_i_ (5.9%) and the normalized SE_i_ (5.8%) (Figure 3). Conversely, the least important parameters were the Freeman–Durden double-bounce (2.1%) and anisotropy (2%). Parameters related to backscattering coefficients were less important (4.3% and 2.4% for VV backscattering and the HV:HH ratio, respectively). For the dual-pol data, the most important parameters were SE and normalized SE, with an importance of 11.5% for both for the ALS-2 L-Band; 13.1% and 12.5%, respectively, for RST-2 and 11.7% and 11.6%, respectively, for S-1. In addition, for the ALS-2 L-Band, SPAN was the most important parameter (11.7%). Conversely, the least important parameter was the HH:HV ratio for the ALS-2 L-Band and RST-2 C-Band (5.6% and 6.4%, respectively) and the difference VV – VH for the S-1 C-Band (6.7%).

Concerning band frequency, in the dual-pol L-Band (Dual-L) configuration, parameters related to backscattering coefficients were less important than polarimetric parameters (mean importance of 6.6% and 10.5%, respectively) (Figure 4). In the dual-pol C-Band (Dual-C) configuration, the ranks of the parameters related to backscattering coefficients were similar for RST-2 and S-1 classification models. For each of them, a backscattering coefficient was important: the HV coefficient for RST-2 and VV coefficient for S-1 (10.4% and 10.7%, respectively). Similarly, the HH:HV and VV:VH ratios were less important (6.4% and 6.7%, respectively). The importance of polarimetric parameters was similar for Dual-C and Dual-L configurations, with SE and SE_i_ being the most important parameters. However, SPAN was the most important parameter for Dual-L but one of the least important for Dual-C.

### 3.2. Contribution of Polarization Mode to Accuracy of Winter Land-Use Classification

**Figure 5:**
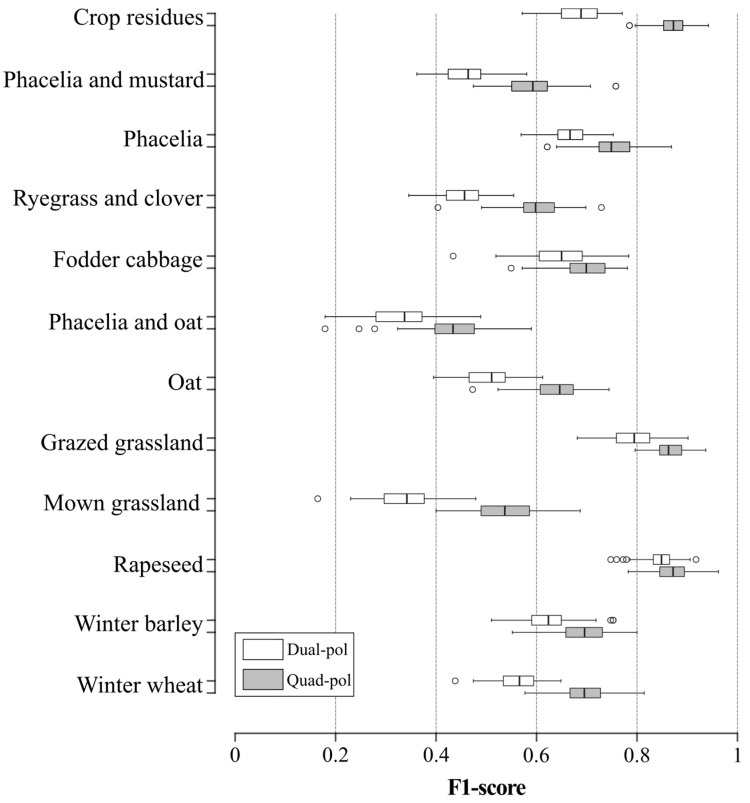
Comparison of classification accuracy of each land-use class between dual and quad polarization (pol) modes. Box-and-whisker plots represent the variation in random forest classification accuracy based on 100 iterations. Whiskers indicate 1.5 times the interquartile range.

The F1-score of land-use classification was higher using the quad-pol dataset (median F1-score 0.69, standard deviation (SD) 0.05; median Kappa 0.68, SD 0.02;) than the dual-pol datasets (median F1-score 0.59, SD 0.05; median Kappa 0.54, SD 0.02) (Figure 5). The superiority of quad-pol mode over dual-pol mode was observed for all winter land-use classes (Figure 5). The largest differences in median F1-score between quad-pol and dual-pol mode concerned the oat (0.64 and 0.50, respectively), ryegrass and clover (0.60 and 0.46, respectively) and phacelia (0.75 and 0.66, respectively) classes. Conversely, the rapeseed class was discriminated slightly better using the quad-pol rather than dual-pol dataset (median F1-score 0.87 and 0.85, respectively). Moreover, Standard Deviation (SD) in F1-score accuracy was slightly lower using the quad-pol rather than dual-pol dataset for fodder cabbage (0.05 and 0.06, respectively) and crop residue (0.03 and 0.04, respectively) classes, but the opposite is true for other classes such as ryegrass and clover (0.05 and 0.04, respectively) (Figure 5).

### 3.3. Contribution of Band Frequency to Accuracy of Winter Land-Use Classification

**Figure 6:**
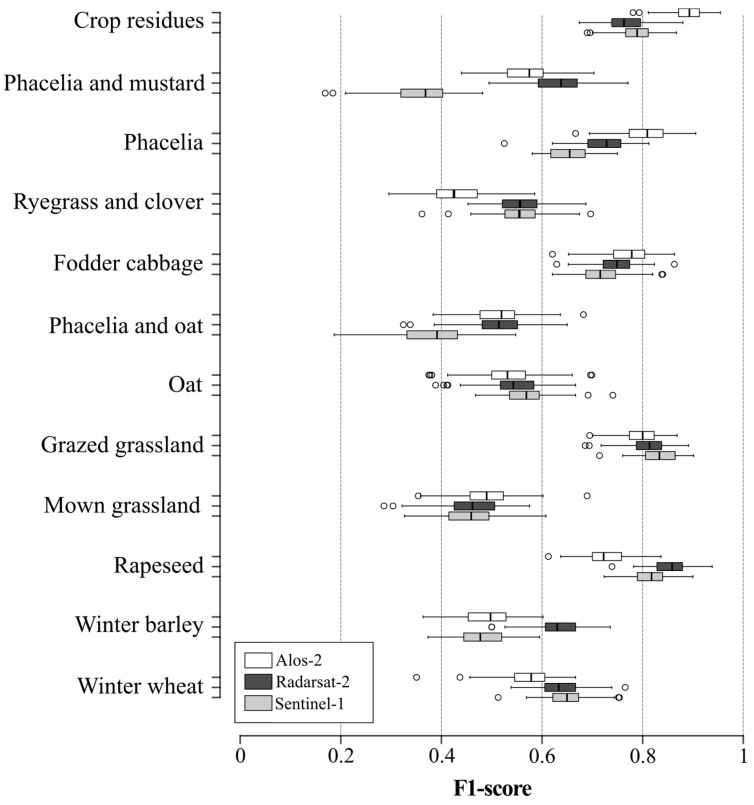
Comparison of classification accuracy of each land-use class among band frequencies. Box-and-whisker plots represent the variation in random forest classification accuracy based on 100 iterations. Whiskers indicate 1.5 times the interquartile range.

The F1-score of land-use classification was slightly higher for the RST-2 C-Band frequency (median F1-score 0.64, SD 0.05; median Kappa 0.64, SD 0.02) than S-1 C-Band frequency (median F1-score 0.61, SD 0.05; median Kappa 0.59, SD 0.02) or the ALS-2 L-Band frequency (median F1-score 0.57, SD 0.05; median Kappa 0.60, SD 0.02) (Figure 6). More precisely, classification accuracy depended on the band frequency and land-use class (Figure 6). F1-score was highest for the C-Band frequency, except for the crop residue, phacelia, fodder cabbage and mown grassland classes, for which the ALS-2 L-Band frequency had the highest accuracy (median F1-score 0.89, 0.80, 0.77 and 0.49, respectively). Classification accuracy using the S-1 parameter dataset was highest for grazed grassland, winter wheat and oat classes (median F1-score 0.83, 0.65 and 0.57, respectively). For the other classes (phacelia and mustard, ryegrass and clover, phacelia and oat, rapeseed and winter barley), F1-score was highest using the RSR-2 parameter dataset. Regardless of the band frequency, the F1-score of the rapeseed, grazed grassland and crop residue classes was higher than those of the other classes (Figure 6). The Standard Deviation in classification accuracy of land-use classes was similar among the ALS-2, RST-2 and S-1 models (0.05, 0.049 and 0.05, respectively).

### 3.4. Contribution of Time-Series Density to Accuracy of Winter Land-Use Classification

**Figure 7:**
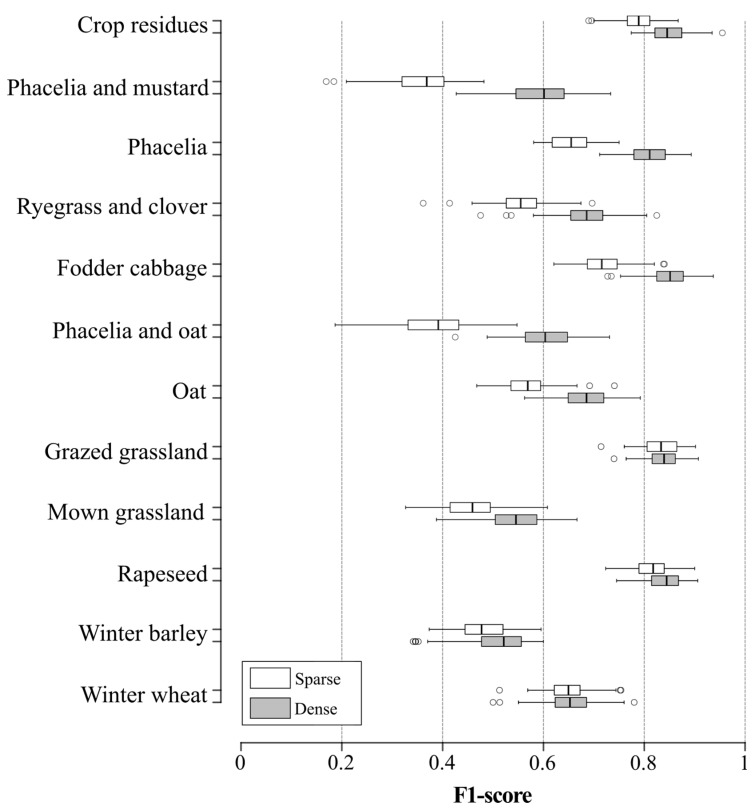
Comparison of classification accuracy of each land-use class between sparse and dense Sentinel-1 time-series. Box-and-whisker plots represent the variation in random forest classification accuracy based on 100 iterations. Whiskers indicate 1.5 times the interquartile range.

**Figure 8:**
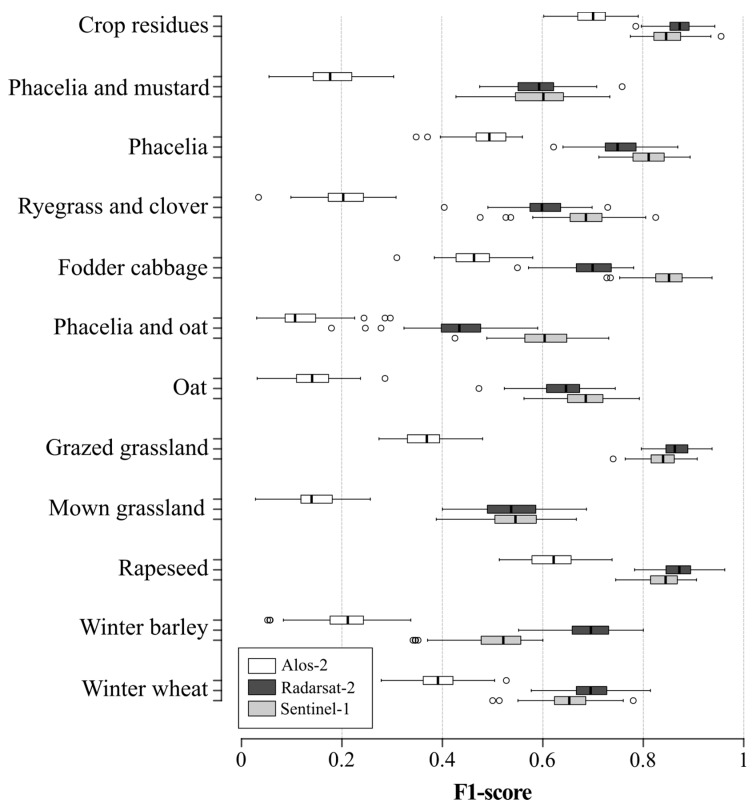
Comparison of classification accuracy of each land-use class among SAR sensors. Box-and-whisker plots represent the variation in RF classification accuracy based on 100 iterations. Whiskers indicate 1.5 times the interquartile range.

The F1-score of land-use classification was higher for the dense S-1 time-series (median F1-score 0.70, SD 0.05; median Kappa 0.69, SD 0.02) than for the sparse S-1 time-series (median F1-score 0.61, SD 0.05; median Kappa 0.60, SD 0.02) (Figure 7). The largest differences between dense and sparse time-series concerned the phacelia and mustard (median F1-score 0.59 and 0.36, respectively), phacelia and oat (median F1-score 0.60 and 0.39, respectively) and phacelia (median F1-score 0.80 and 0.66, respectively) classes. Moreover, the variance in classification accuracy was slightly lower for the dense rather than sparse S-1 time-series of the phacelia and oat (SD 0.06 and 0.07, respectively) and grazed grassland (SD 0.03 and 0.04, respectively) classes (Figure 8). Conversely, the variance in classification accuracy in sparse and dense time-series of some winter land-use classes, such as winter wheat (SD 0.04 and 0.05, respectively) and winter barley (SD 0.05 and 0.06, respectively), was higher using the dense time-series.

### 3.5. Definition of the Best SAR Configuration

The F1-score of land-use classification was slightly higher using the S-1 dense time-series (median F1-score 0.70, SD 0.00; median Kappa 0.69, SD 0.02) than using the RST-2 quad-pol (median F1-score 0.69, SD 0.05; median Kappa 0.68, SD 0.02) or ALS-2 original dual-pol (median F1-score 0.29, SD 0.05; median Kappa 0.32, SD 0.02) time-series (Figure 8). More precisely, classification accuracy depended on the sensor and land-use class (Figure 8). The F1-score was higher with the S-1 model, except for the winter wheat, winter barley, rapeseed, grazed grassland and crop residue classes, for which the RST-2 model had higher accuracy. The accuracy of the S-1 model was slightly higher (by 0.01 in the F1-score 1%) than that of RST-2 for the phacelia and mustard and mown grassland classes, while that of the ALS-2 model was always lower than those of S-1 and RST-2. Finally, the variance in classification accuracy was similar among the S-1, RST-2 and ALS-2 models (SD 0.05, 0.05 and 0.05, respectively).

### 3.6. Spatial Distribution of Winter Land-Use Classes

**Figure 9:**
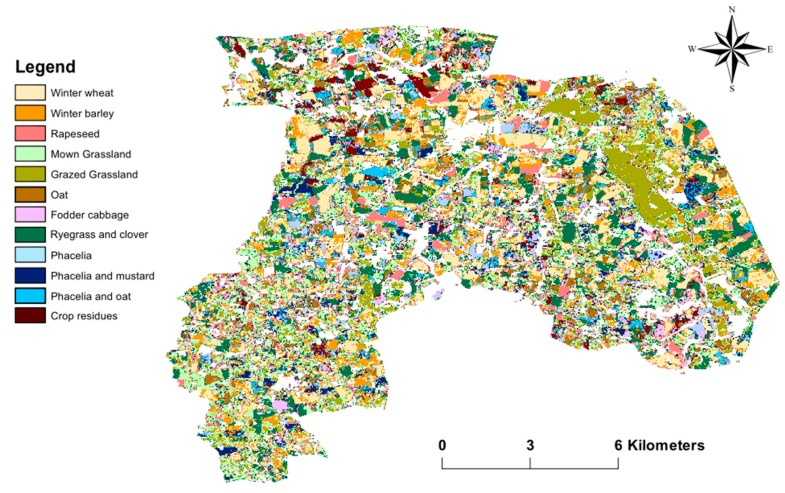
Map of winter land-use classes obtained using a parameter dataset derived from the Sentinel-1 dense time-series. Classification was performed using the random forest algorithm.

Winter land-use classes were mapped at the 1:100 000 scale using the best classification model: the RF classification using the SAR parameter dataset extracted from S-1 dense time-series images. While grasslands were classified well (F1-score, 0.71), some misclassification errors and artifacts occurred, mainly between catch-crop classes, such as oat and phacelia and oat classes (Table 3). In general, catch crops and winter crops were located on the largest fields, while grasslands were located on the smallest ones (Figure 9).

## 4. Discussion

This study aimed to evaluate and compare the value of multi-temporal ALS-2, RST-2 and S-1 data for monitoring winter land-use in agricultural areas. Winter land-use classes, which depend on vegetation characteristics and phenology, can be discriminated using the specific properties of SAR sensors to identify crops [5,51,52]. A two-step method was applied to the study site. First, SAR parameters were derived from RST-2, S-1, and ALS-2 time-series, and one quad-pol and six dual-pol parameter datasets with different spatial resolutions and densities were calculated from these time-series. Then, land use was classified using the RF algorithm with each of these seven SAR parameter datasets to determine the most suitable SAR configuration for identifying winter land-use.

### 4.1. Which SAR Configuration for Mapping Winter Land-Use?

Our study took advantage of the potential of SAR sensor characteristics to identify winter land-use types. The physical properties and specific resolutions (spatial, temporal and frequency) of ALS-2, RST-2 and S-1 sensors allowed us to discriminate winter land-use classes in our study area, including areas where the agricultural landscape was fragmented. However, some SAR configurations appear to be more effective than others.

Results highlight that the C-Band outperformed the L-Band, the quad-polarization mode outperformed the dual-polarization mode and S-1 dense time-series outperformed RST-2 and ALOS-2 time-series. Overall, the dense S-1 time-series was the most suitable SAR configuration data for winter land-use identification, with classification accuracy slightly higher than those of the others. This result is consistent with other studies that have reported the added value of S-1 data for mapping cropland [44,53] or identifying and characterizing crop phenology [27]. Nevertheless, the quad-pol C-Band RST-2 dataset also showed potential to identify winter land-use, with accuracies similar to those obtained using the dense dual-pol C-Band S-1 dataset. This result is consistent with the studies of [54,55], which also showed the potential of polarimetric RST-2 data for monitoring and classifying crops. Conversely, the dual-pol L-Band ALS-2 dataset appeared to be of little use for identifying and mapping land-use in winter, except for certain classes such as crop residues or phacelia and mustard. These results add information to the research to date, which has demonstrated the ability of the L-Band to classify crops in summer [17,56]. However, these results should be interpreted with caution because, as shown by the results obtained with S-1 images, time-series density has a significant impact on the identification of winter land-use classes, as shown by [57]. Thus, if the classification score obtained with the ALS-2 time-series is lower than that obtained with the RST-2 and S-1 sparse time-series (F1-score 0.57 vs 0.61 and 0.64, respectively), it should be noted that the ALS-2 time-series had the least number of images (6 against 10 and 8 for RST-2 and S-1, respectively). In addition, the difference between the incidence angles of the images, although small (35° for RTS-2 and 40° for S-1 and ALS-2), can have an impact on the accuracy of the results, as noted by [58,59].

Results indicate that SE and SPAN were the most important parameters, while difference and ratio parameters had low importance. These results are consistent with the research conducted to date. [60] demonstrated the importance of SE in studying land use and land cover. Some studies also demonstrated the ability of SE to characterize the canopy of vegetated areas [61]. Likewise, many studies (e.g., [62], [63]) have described the ability of SPAN parameters to classify crops.

The best accuracy, obtained using the dense dual-pol C-Band S-1 dataset (OA 72%), demonstrated the limits of using one SAR dataset alone to identify winter land-use accurately. Nevertheless, our results demonstrate the ability of other band frequencies to discriminate land-use classes. For example, ALS-2 data appear to be useful for classifying crop residue and phacelia and mustard classes. Similarly, RST-2 data appear to be useful for classifying rapeseed and grazed grassland classes. These results highlight the utility of combining SAR parameters. For example, [17] demonstrated the utility of combining C-Band and L-Band SAR data for classifying crops. Hence, use of combined ALS-2, RST-2 and S-1 SAR datasets should be considered to obtain a denser time-series to discriminate between land-use types better.

### 4.2. Advantages and Disadvantages of the Classification Approach

Results demonstrated the potential of this classification approach based on the RF algorithm, although the highest F1-score achieved was 0.70. Some studies based on SAR data classified using the RF algorithm also highlighted this complexity in identifying winter crops, unlike for annual crops such as maize [5,27]. Moreover, the results also confirm the ability of SAR data to classify crop residues, as some previous studies have shown [19,64].

Several other approaches can be considered. In this study, the term time-series refers to a set of images acquired during a given period, and each acquisition was processed as an independent image without capturing properties of time-series data. New approaches that take time explicitly into account such as TWDTW (Time-Weighted Dynamic Time Warping) algorithm have proven to be very effective in solving complex classification problems. Although few studies of winter land-use classification have been performed, [28] for example showed the utility of deep learning for classifying vegetation quality (cover density) in winter using S-1 data, with preliminary results achieving a Kappa index of 0.98. However, these approaches require high-performance calculations and need to run hyperparameter searches, adjustments and tests, which need further investigations that will be done in future work. Furthermore, as mentioned, the results highlighted the value of specific radar parameters in the L- and C-Bands for discriminating winter land-use classes. These results highlight the utility of approaches that combine data to classify crops, as demonstrated in certain studies [17,18].

## 5. Conclusions

This study evaluated advantages of using S-1, RST-2 and/or ALS-2 time-series to determine the best SAR configuration to identify winter land-use classes accurately using the RF algorithm. Several SAR configurations were tested to discriminate land-use types during winter using the RF algorithm; to our knowledge, this is the first time such a study has been undertaken. Results show that the best SAR configuration was the dense dual-pol C-Band S-1 time-series, although RST-2 and ALS-2 time-series provided useful information about vegetation cover. Finally, our results demonstrated the limits of using one SAR dataset alone to identify winter land use accurately, the highest F1-score reaching only 0.70. Thus, future research could study the utility of combining SAR parameters or using new classification approaches based on deep learning to improve the accuracy of classifying land-use types in winter. Better understanding of SAR signal behaviors of agricultural practices and environmental conditions could also help to identify winter land use, which has important implications for developing sustainable agriculture.

## Figures and Tables

**Table 1:** Land-use classification.

Winter Land Use Type	Main Crops
Winter crops	Winter wheat
	Winter barley
	Rapeseed
Grasslands	Mown grasslands
	Grazed grasslands
Catch crops	Oat
	Fodder cabbage
	Ryegrass and clover
	Phacelia
	Phacelia and mustard
	Phacelia and oat
Crop residues	Maize stalks

**Table 2:** Characteristics of the RADARSAT-2, Sentinel-1 and Advanced Land Observing Satellite 2 (ALOS-2) images used in the study. Dates in bold text for Sentinel-1 images indicate those with sparse time-series, while asterisks indicate those with dense time-series.

	RADARSAT-2	Sentinel-1	ALOS-2
**Dates** **(M-D-Y)**	10-23-2016 11-16-2016 12-10-2016 01-03-2017 01-27-2017 02-20-2017 03-16-2017 04-09-2017 05-03-2017 05-27-2017	**08-25-2016** 09-18-2016* 09-30-2016* 10-12-2016* 10-24-2016* 11-05-2016* 11-17-2016* 11-29-2016* 12-11-2016* 12-23-2016* 01-04-2017* 01-16-2017* 01-28-2017* 02-09-2017* 02-21-2017* 03-05-2017* 03-17-2017* 03-29-2017* 04-10-2017* 04-22-2017* 05-04-2017* 05-16-2017	01-04-2017 02-04-2017 03-06-2017 04-15-2017 05-13-2017 06-10-2017
**Ground** **Resolution**	8.2 m	2.3 m	1.4 m
**Azimuth** **Resolution**	4.7 m	13.9 m	1.9 m
**Polarization**	Quad (HH-VV-HV-VH)	Dual (VV – VH)	Dual (HH-HV)
**Frequency**	C-Band	C-Band	L-Band
**Mode**	Fine Quad Polarization (SLC)	Interferometric wide (SLC)	Spotlight (SLC)
**Incidence Angle**	35° (right descending)	31° to 46° (right descending)	40° (left ascending)
**Coverage**	18 km × 25 km	>250 km × 100 km	25 km × 25 km

**Table 3:** Confusion matrix of winter land-use obtained from RF classification using the SAR parameter dataset extracted from the S-1 dense time-series dataset.

Land Use	1	2	3	4	5	6	7	8	9	10	11	12	Commission Error (%)
1: Winter wheat	32	1	4	1	0	0	2	0	1	0	2	0	74.4
2: Winter barley	11	22	0	6	2	2	4	0	1	0	5	0	41.5
3: Rapeseed	1	3	49	2	1	3	2	2	1	1	0	1	74.2
4: Mown grasslands	0	3	2	28	7	0	1	0	0	2	1	2	60.9
5: Grazed grasslands	0	0	0	0	44	0	1	2	1	0	0	0	91.7
6: Oat	0	2	0	5	0	33	8	0	1	4	2	1	58.9
7: Phacelia and oat	0	1	0	2	0	4	26	0	5	3	3	0	59.1
8: Fodder cabbage	1	0	1	3	0	1	1	41	2	0	2	1	77.4
9: Ryegrass and clover	1	1	1	1	2	2	0	4	36	0	1	0	73.5
10: Phacelia	0	0	1	0	0	1	5	0	0	41	4	0	78.9
11: Phacelia and mustard	1	0	0	0	0	1	0	0	0	0	34	1	91.9
12: Crop residues	0	0	0	0	0	3	0	0	2	0	4	44	83.0
Omission error (%)	68.1	66.7	84.5	58.3	78.6	66.0	52.0	83.7	72.0	80.4	58.6	88.0	71.7
